# Modeling framework for isotopic labeling of heteronuclear moieties

**DOI:** 10.1186/s13321-017-0201-7

**Published:** 2017-03-04

**Authors:** Mark I. Borkum, Patrick N. Reardon, Ronald C. Taylor, Nancy G. Isern

**Affiliations:** 1 Environmental Molecular Sciences Laboratory, Pacific Northwest National Laboratory, 3335 Innovation Boulevard, Richland, WA 99354 USA; 2 Biological Sciences Division, Pacific Northwest National Laboratory, 3335 Innovation Boulevard, Richland, WA 99354 USA

**Keywords:** Isotopic labeling, Isotopomers, Cumomers, Elementary metabolite units, Metabolic engineering

## Abstract

**Background:**

Isotopic labeling is an analytic technique that is used to track the movement of isotopes through reaction networks. In general, the applicability of isotopic labeling techniques is limited to the investigation of reaction networks that consider homonuclear moieties, whose atoms are of one tracer element with two isotopes, distinguished by the presence of one additional neutron.

**Results:**

This article presents a reformulation of the modeling framework for isotopic labeling, generalized to arbitrarily large, heteronuclear moieties, arbitrary numbers of isotopic tracer elements, and arbitrary numbers of isotopes per element, distinguished by arbitrary numbers of additional neutrons.

**Conclusions:**

With this work, it is now possible to simulate the isotopic labeling states of metabolites in completely arbitrary biochemical reaction networks.

## Introduction

Isotopic labeling is an analytic technique that is used to track the movement of isotopes through reaction networks. First, specific atoms of the reagent moieties are replaced with detectable, “labeled” isotopic variants. Then, after the reactions have been allowed to proceed, the position and relative abundance of labeled isotopic atoms are determined by experiment. Subsequent analysis of the measured quantities elucidates the characteristics of the reaction network, e.g., the rate constants of the reactions.

In general, the applicability of isotopic labeling techniques is limited to the investigation of reaction networks that consider homonuclear moieties, whose atoms are of one isotopic tracer element with two isotopes, distinguished by the presence of one additional neutron.

The contribution of this article is a reformulation of the modeling framework for isotopic labeling, generalized to arbitrarily large, heteronuclear moieties, arbitrary numbers of isotopic tracer elements, and arbitrary numbers of isotopes per element, distinguished by arbitrary numbers of additional neutrons.

## Background

The first group to give a partial solution to the problem of the representation of isotopic labeling states of arbitrary moieties was Malloy et al. [1].

Representing isotopic labeling states of carbon atoms in backbones of metabolites as vectors of Boolean truth values, which they referred to as “isotopomers” (a contraction of the term “isotopic isomers”), using 0 and 1 to denote, respectively, ^12^ ${\textsf{C}}$-unlabeled and ^ 13^ ${\textsf{C}}$-labeled atoms, they showed that relative abundances of metabolites in biological systems could be calculated using nonlinear functions of relative abundances of isotopomers, which they referred to as “isotopomer fractions.”

For example, a metabolite of two carbon atoms has $2^{2} = 4$ isotopomers, 00, 01, 10 and 11; and hence, the isotopic labeling state of the metabolite is given by 4 isotopomer fractions.

Aside from being nonlinear, Malloy et al.’s construction suffers from the fact that an exponential number of isotopomer fractions are required in order to determine the isotopic labeling state of any given metabolite.

Wiechert et al. [2] noted that, by the nature of the problem, isotopic labeling states of biochemical reaction network substrates are wholly determined by specific subsets of carbon atoms; and therefore, that isotopic labeling states of the complement of each subset can be omitted, incurring no loss of information.

Representing isotopic labeling states of carbon atoms in backbones of metabolites as vectors of placeholder variables, which they referred to as “cumomers” (a contraction of the term “cumulative isotopomers”), using 1 and *x* to denote, respectively, determinate (^13^ ${\textsf{C}}$-labeled) and indeterminate (^12^ ${\textsf{C}}$-unlabeled or ^ 13^ ${\textsf{C}}$-labeled) isotopic labeling states, with a total ordering given by $x < 1$, they showed that Malloy et al.’s construction could be reformulated as a cascade system of linear functions of relative abundances of cumomers, which they referred to as “cumomer fractions,” with the original construction being recovered via a “suitable variable transformation” [2, Eq. 7] in the form of an invertible square matrix.

For example, a metabolite of two carbon atoms has $2^{2} = 4$ cumomers, *xx*, *x*1, 1*x* and 11; and hence, the isotopic labeling state of the metabolite is given by 4 cumomer fractions.

Antoniewicz et al. [3] shed new light on this subject. Manipulating isotopic labeling states of specific subsets of carbon atoms as aggregations, rather than as singletons, they showed that, under certain conditions, Wiechert et al.’s cascade system could be optimized using graph-theoretic methods, e.g., vertex reachability analysis, edge smoothing and Dulmage–Mendelsohn decomposition, thereby significantly reducing the total number of system variables.

Representing isotopic labeling states of carbon atoms in backbones of metabolites as mass distributions, which they referred to as “Elementary Metabolite Units (EMU),” they showed that every EMU has a unique factorization, and that the mass distribution of a given EMU is equal to the vector convolution of the mass distributions of its proper factors. Moreover, they showed that, in a given mass distribution, the mass fraction corresponding to the greatest number of mass shifts is equivalent to a specific cumomer fraction.

Antoniewicz et al.’s work provided the foundation for a whole host of important biological investigations [4–6], and inspired many software implementations [7–10]. Even so, the cases not solved by Antoniewicz et al. merit attention for four reasons. First, the subject is very closely tied to the concept of isotopic labeling, and can thus serve to bring greater clarity and determinacy to its mathematical formulation. In this respect, treatment of the subject possesses an immediate interest. Second, the EMU method suffers from the same system variable explosion issue as the isotopomer and cumomer methods that preceded it. Is this issue intrinsic to the problem, i.e., unavoidable, or simply, difficult to mitigate? Third, Antoniewicz et al. give neither a rigorous proof of the correctness of the EMU method, nor a derivation of its construction from prior research. Why does the EMU method appear to work? Is it the most effective decomposition that can be “done” to a model of a given biological system? Fourth, and finally, the EMU method has thus far been demonstrated for homonuclear systems only; specifically, those containing carbon atoms, where unlabeled and labeled variants of isotopic tracer elements are distinguished by the presence of one additional neutron. Can the EMU method be generalized to arbitrary, heteronuclear systems?

At the conclusion of their essay, Antoniewicz et al. promise to return to these cases later; but, thus far, this promise has gone unfulfilled. Nor do the works of Srour et al. [11] fill this gap. Their essays, which are contemporaneous with those of Antoniewicz et al., develop a counterpart to the EMU method, where system variables are combinations of flux variables and cumomer fractions, which they refer to as “fluxomers.”

While the fluxomer method does enable a reformulation of the flux balance equation that, it is claimed, has greater numerical precision and does not require matrix inversion for its solution, it is, in actuality, a method for constructing a specific matrix in a single pass, which, with some algebraic manipulation, can be shown to be equivalent to the EMU method for certain biochemical reaction networks. Specifically, the fluxomer method involves the construction of the directed line graph for each EMU reaction network (the new graph that represents the adjacencies between the edges of the original graph). Furthermore, the software implementation of the fluxomer method is, in general, far more complex than that of the EMU method, limiting its widespread adoption.

The most recent work in this area, by Nillson and Jain [12], gives a construction for the representation and manipulation of “multivariate mass isotopomer distributions” of heteronuclear moieties, which they demonstrate for moieties of carbon and nitrogen atoms. The key advantage of their approach is that the probabilities associated with each combination of the contribution of each isotopic tracer element are uniquely identifiable. The disadvantage of their approach, however, is that it requires the use of *k*-dimensional vectors, where *k* is the number of isotopic tracer elements, increasing the complexity of the software implementation. In the discussion of their essay, the authors claim that “the cumomer framework can be easily extended along the same lines;” however, they give neither a construction of multivariate cumomers nor a method of conversion to multivariate mass distributions. Moreover, their method is only demonstrated for isotopic tracer elements with two isotopic labeling states per atom: unlabeled or labeled.

In summary: Malloy et al. represented isotopic labeling states of carbon atoms in backbones of metabolites as probability distributions of configurations, obtaining a nonlinear formulation of the flux balance equation. Wiechert et al. omitted specific isotopic labeling states, obtaining a cascade system. Antoniewicz et al. manipulated aggregations of isotopic labeling states, represented as mass distributions, yielding an optimization; and Srour et al. gave an algebraically equivalent reformulation. Nillson and Jain used multidimensional vectors to represent mass distributions of heteronuclear moieties, but did not give a construction for isotopomers or cumomers. As things stand, however, representation of and conversion between isotopic labeling states of metabolites in completely arbitrary biological systems is not possible.

## Results

### Definitions

The indeterminacy which still prevails on a number of fundamental points in the theory of isotopic labeling compels us to make some prefatory remarks about the concept of an isotopic labeling state and the scope of its validity.

For this investigation, unless otherwise stated, a moiety is any set of atoms, not necessarily connected via chemical bonds, nor of the same metabolite. Furthermore, we take as an axiom that the number of nucleons in an atomic nucleus is quantized, i.e., is a non-negative integer.

First and foremost, what do we understand by the concept of an isotopic labeling state?

Representations of states of moieties of isotopic atoms are isomorphic to logical conjunctions of assertions of both the proton and neutron numbers of each atom, e.g., “the first atom has 1 proton and 0 neutrons, the second atom has 6 protons and 7 neutrons, etc.” In this way, the characteristics of each isotopic atom are completely specified; and hence, the representation is an unambiguous description of the isotopic labeling state of the given moiety.

However, if there exists a mapping $f\,{:}\,{\mathbb {Z}} \rightarrow {\mathbb {Z}}$ from proton to *least* neutron number, e.g., $f\left( {1} \right) = 0, f\left( {6} \right) = 6$, etc., where ${\mathbb {Z}}$ denotes the set of integers, then representations of states of moieties are isomorphic to logical conjunctions of assertions of both the proton and *additional* neutron numbers, i.e., mass shifts, of each atom, e.g., “the first atom has 1 proton and 0 additional neutrons, the second atom has 6 protons and 1 additional neutron, etc.” In this way, the characteristics of each isotopic atom are still completely specified; but, the total number of system variables per moiety, required to represent the mass distribution, is reduced to the successor of the greatest additional neutron number.

Let *m* be a non-negative integer that denotes the greatest neutron number (either directly or, assuming the existence of a mapping from proton to least neutron number, indirectly), then the set of determinate isotopic labeling states of a given isotopic atom of a given moiety, denoted ${\text {St}}_{m}$, is the set of integral members of the closed interval $\left[ {0,\,m} \right]$

1 $$\begin{aligned} {\text {St}}_{m} \overset{\text {def}}{=} \left\{ {0,\,1,\,\ldots ,\,m } \right\} . \end{aligned}$$

Let *n* and *m* be non-negative integers that denote, respectively, the number of isotopic atoms and greatest neutron number, then the set of isotopomers of a given moiety, denoted ${\text {Isotopomer}}_{n,\,m}$, is the set of length-*n* vectors of members of the set ${\text {St}}_{m}$

2 $$\begin{aligned} {\text {Isotopomer}}_{n,\,m} \overset{\text {def}}{=} \left\{ {\left\{ { a_{1},\,a_{2},\,\dots ,\,a_{n} } \right\} \vert \ \forall \ i : \left( {i \in \left[ {1,\,n} \right] } \right) \wedge \left( {a_{i} \in {\text {St}}_{m}} \right) } \right\} , \end{aligned}$$

and the set of cumomers of a given moiety, denoted ${\text {Cumomer}}_{n,\,m}$, is the set of sequences of members of the set ${\text {St}}_{m} \cup \left\{ {\top } \right\}$

3 $$\begin{aligned} {\text {Cumomer}}_{n,\,m}\overset{\text {def}}{=} \left\{ { \left( {a_{1},\,a_{2},\,\dots ,\,a_{n},\,a_{n + 1},\,\ldots } \right) \vert \ \forall \ i : \left( {i \in {\mathbb {Z}}} \right) \wedge \left( { a_{i} \in \left\{ { \begin{array}{lll} {\text {St}}_{m} \cup \left\{ {\top } \right\} &\quad {\text {if}} & i \le n\\ \left\{ {\top } \right\} &\quad {\text {if}} & i > n\\ \end{array} } \right. } \right) } \right\} , \end{aligned}$$

where $\top$ denotes the indeterminate isotopic labeling state, the logical disjunction of every determinate isotopic labeling state (viz., logical true).

Importantly, while an isotopomer is a representation of the isotopic labeling state of a given moiety in the context of itself, a cumomer is a representation of the isotopic labeling state of a given moiety in the context of a larger, virtual moiety of a countably infinite number of atoms, whose isotopic labeling states are mutually independent, where the isotopic labeling states of non-local atoms are, by definition, indeterminate (as displayed by the quantification of atom indices over the nonzero, positive members of ${\mathbb {Z}}$). It is this essential difference that enables the formulation of the flux balance equation as a cascade system.

Let *n* and *m* be non-negative integers that denote, respectively, the number of isotopic atoms and the greatest neutron number, then the set of cumomers of a given moiety can be exhaustively partitioned into a set of $2^{n}$ mutually disjoint subsets, denoted ${\text {EMU}}_{n,\,m}$, where the isotopic labeling states of local atoms in each subset of cumomers, denoted ${\text {EMU}}_{n,\,m,\,N}$, are determinate for the members of a member *N* of the power-set of the set of atoms of the given moiety

4 $$\begin{aligned}&{\text {EMU}}_{n,\,m}\overset{\text {def}}{=}\left\{ {\text {EMU}_{n,\,m,\,N} \vert \ \forall \ N : \left( {N \in {\mathbb {P}}\left( {\left[ {1,\,n} \right] } \right) } \right) } \right\} \nonumber \\&{\text {EMU}}_{n,\,m,\,N} \overset{\text {def}}{=} \left\{ { a\ \vert \left( {a \in {\text {Cumomer}}_{n,\,m}} \right) \wedge \left( { \forall \ i : \left( {i \in \left[ {1,\,n} \right] } \right) \wedge \left( { a_{i} \in \left\{ { \begin{array}{ccc} {\text {St}}_{m} & {\text {if}} & i \in N\\ \left\{ {\top } \right\} & {\text {if}} & i \not \in N\\ \end{array} } \right. } \right) } \right) } \right\} , \end{aligned}$$

where ${\mathbb {P}}$ denotes the power-set function.

### Representation as Boolean functions

Isotopomer and cumomer fraction vectors for uni- and multi-atomic EMUs are, by definition, discrete probability distributions of the isotopic labeling state of the given moiety. Interpreted as Boolean functions, isotopomer and cumomer fraction vectors are “multiplied” using the Cartesian product under multiplication.

Let *n* and *m* be non-negative integers that denote, respectively, the number of isotopic atoms and the greatest neutron number, then the set of isotopomer fractions of a given moiety, corresponding to the set ${\text {Isotopomer}}_{n,\,m}$, represented as a vector of $\left( {m + 1} \right) ^{n}$ entries, each giving the functional value of the corresponding minterm, is a completely specified, Boolean function of *n* variables, with $m + 1$ values per variable. The sum of the set of isotopomer fractions of a given moiety is, therefore, =100%.

Let *n* and *m* be non-negative integers that denote, respectively, the number of isotopic atoms and the greatest neutron number, and $N \in {\mathbb {P}}\left( {\left[ {1,\,n} \right] } \right)$ be a subset of atoms with determinate isotopic labeling states, then any (mutually disjoint) subset of cumomer fractions of a given moiety, corresponding to the set ${\text {EMU}}_{n,\,m,\,N}$, represented as a vector of $\left( {m + 1} \right) ^{\left| {N} \right| }$ entries, each giving the functional value of the corresponding minterm, is a completely specified, Boolean function of $\left| {N} \right|$ variables, with $m + 1$ values per variable. The sum of a (mutually disjoint) subset of cumomer fractions of a given moiety is, therefore, =100 %.

Let *n* and *m* be non-negative integers that denote, respectively, the number of isotopic atoms and the greatest neutron number, then the set of cumomer fractions of a given moiety, corresponding to the set ${\text {Cumomer}}_{n,\,m}$, represented as a vector of $\left( {m + 2} \right) ^{n}$ entries, each giving the functional value of the corresponding minterm, is a completely specified, Boolean function of *n* variables, with $m + 2$ values per variable (the extra value being the intermediate isotopic labeling state). The sum of the set of cumomer fractions of a given moiety is, therefore, ${=}2^{n} \times 100\%$.

For the above to hold in situations where isotopic tracer elements have different numbers of isotopes, distinguished by different numbers of additional neutrons, it is necessary to assume that surplus isotopic labeling states are undetectable, i.e., that the probability of their detection is =0%. Given this assumption, isotopomers and cumomers that include surplus isotopic labeling states are also undetectable, i.e., the corresponding isotopomer and cumomer fractions are also =0%.

### Representation as mass distributions

Cumomer fraction vectors for uni-atomic EMUs are, by definition, probability mass functions that characterize discrete probability distributions of the mass of the given atom, i.e., mass distributions. Interpreted as mass distributions (instead of Boolean functions), cumomer fraction vectors are “multiplied” using vector convolution (instead of the Cartesian product under multiplication).

### Representation as arbitrary monoids

Both interpretations induce a commutative monoid structure on the set of EMUs, where the left- and right-identity of the monoidal product is the interpretation of the trivial EMU, corresponding to the “empty moiety” of zero atoms, denoted ${\text {EMU}}_{n,\,m,\,\emptyset }$, where $\emptyset$ denotes the empty set.

What other interpretations are possible for uni-atomic EMUs? Under what circumstances are they valid?

A homomorphism $f\,{:}\,A \rightarrow B$ is a function between two sets *A* and *B*, such that

5 $$\begin{aligned} f\left( { \mu _{A}\left( { a_{1},\,a_{2},\,\dots ,\,a_{n} } \right) } \right) = \mu _{B}\left( { f\left( {a_{1}} \right) ,\,f\left( {a_{2}} \right) ,\,\ldots ,\,f\left( {a_{n}} \right) } \right) \end{aligned}$$

holds, for the variadic functions $\mu _{A}$ and $\mu _{B}$, and for all elements $a_{1},\,a_{2},\,\dots ,\,a_{n} \in A$.

Let *n* and *m* be non-negative integers that denote, respectively, the number of isotopic atoms and the greatest neutron number, and $N \in {\mathbb {P}}\left( {\left\{ {1,\,2,\,\ldots ,\,n} \right\} } \right)$ be a subset of atoms with determinate isotopic labeling states, then the function $f\,{:}\,\left\{ {{\mathbb {Z}}} \right\} \rightarrow S$ is a homomorphism between the domain, the set of (mutually disjoint) subsets of atoms, and the codomain *S*, where $\mu _{\left\{ {{\mathbb {Z}}} \right\} }$ and $\mu _{S}$ are, respectively, set union and the monoidal product.

Interpretations of uni-atomic EMUs are valid, therefore, if and only if:

- The codomain *S* is isomorphic to the set of vectors; whose lengths, non-negative integers, are given by a well-defined function of $\left| {N} \right|$ and *m*; and,
- The codomain *S* is a commutative monoid.

Hence, given *n* and *m*, the function $f\left( {N} \right) = {\text {EMU}}_{n,\,m,\,N}$ is a valid homomorphism, with the codomain being interpreted as either the set of mass distributions or the set of Boolean functions. Moreover, the function $f\left( {N} \right) = \left| N \right|$ is also a valid homomorphism, with the codomain being the set of non-negative integers under addition.

Notice that, if we follow Wiechert et al.’s advice and partition the members of the codomain *S* by the number of isotopic atoms, then, by definition, the vector representations of the members of each partition have the same length. Therefore, any valid interpretation can be used in flux balance equations if the corresponding vector representations are transposed (from column to row vectors).

### Conversion of isotopomer and cumomer fractions

 Let *n* and *m* be non-negative integers that denote, respectively, the number of isotopic atoms and the greatest neutron number, then conversion from isotopomer fraction vectors to cumomer fraction vectors is “done” using the rectangular matrix6where $\otimes$ denotes the Kronecker product, and ${\mathbb {I}}_{k}$ denotes the identity matrix of dimension $k \times k$.

Exemplar matrices for small values of *n* and *m* are given in Table 1.

**Table 1:** Matrices for conversion of isotopomer and cumomer fraction vectors

$\overline{\mathbf{IC }}_{n,m}$	$n=0$	$n=1$	$n=2$
$m=0$	$$\begin{bmatrix} 1 \end{bmatrix}$$	$$\begin{bmatrix} 1 \end{bmatrix}$$	$$\begin{bmatrix} 1 \end{bmatrix}$$
$m=1$	$$\begin{bmatrix} 1 \end{bmatrix}$$	$$\begin{bmatrix} 1&0\\ 0&1\\ 1&1\\ \end{bmatrix}$$	$$\begin{bmatrix} 1&0&0&0\\ 0&1&0&0\\ 1&1&0&0\\ 0&0&1&0\\ 0&0&0&1\\ 0&0&1&1\\ 1&0&1&0\\ 0&1&0&1\\ 1&1&1&1\\ \end{bmatrix}$$
$m=2$	$$\begin{bmatrix} 1 \end{bmatrix}$$	$$\begin{bmatrix} 1&0&0\\ 0&1&0\\ 0&0&1\\ 1&1&1\\ \end{bmatrix}$$	$$\begin{bmatrix} 1&0&0&0&0&0&0&0&0\\ 0&1&0&0&0&0&0&0&0\\ 0&0&1&0&0&0&0&0&0\\ 1&1&1&0&0&0&0&0&0\\ 0&0&0&1&0&0&0&0&0\\ 0&0&0&0&1&0&0&0&0\\ 0&0&0&0&0&1&0&0&0\\ 0&0&0&1&1&1&0&0&0\\ 0&0&0&0&0&0&1&0&0\\ 0&0&0&0&0&0&0&1&0\\ 0&0&0&0&0&0&0&0&1\\ 0&0&0&0&0&0&1&1&1\\ 1&0&0&1&0&0&1&0&0\\ 0&1&0&0&1&0&0&1&0\\ 0&0&1&0&0&1&0&0&1\\ 1&1&1&1&1&1&1&1&1\\ \end{bmatrix}$$

Non-square, rectangular matrices are, of course, non-invertible; however, isomorphisms between the set of isotopomers and certain subsets of cumomers can be defined, witnessed by a family of square matrices, as we will now show.

Let *m* be a non-negative integer that denotes the greatest neutron number, and $s \in {\text {St}}_{m}$ be a determinate isotopic labeling state, then the set of *punctured*, determinate isotopic labeling states of an isotopic atom of a given moiety with respect to *s* is the set ${\text {St}}_{m} \setminus \left\{ {s} \right\}$. Removal of the $\left( {s + 1} \right)$th row of the rectangular matrix $\overline{\mathbf{IC }}_{1,\,m}$, followed by the repeated Kronecker product, always yields an invertible matrix. In fact, for a given *n* and *m*, the product of any two of these invertible matrices is an injective matrix (the utility of which we have not yet established).

Why is this the case? For every determinate isotopic labeling state *s*, there exists a set of punctured, determinate isotopic labeling states, and a corresponding set of punctured cumomers (the term “punctured” referring to the exclusion of any one member of the set). The set of punctured cumomers is, therefore, the logical conjunction-exclusive disjunction expansion of the set of isotopomers with respect to *s*; or, in coding theory parlance, the Reed–Muller spectral domain [13, 14] of the set of isotopomers with respect to *s*. The square matrices are, therefore, the Reed–Muller transform matrices.

Note that the set of “cumomers” described by Wiechert et al. is the set of punctured cumomers with respect to *s* = 0.

### Conversion of isotopomer and mass fractions

Let *n* and *m* be non-negative integers that denote, respectively, the number of isotopic atoms and the greatest neutron number, then conversion from isotopomer fraction vectors to mass fraction vectors is “done” using the rectangular matrix

7 $$\begin{aligned} \overline{\mathbf{IM }}_{n,\,m} \overset{\text {def}}{=} \prod _{i =1}^{n}{ {\text {interspersecols}}\left( {{\mathbb {I}}_{m + 1},\,\left( {m + 1} \right) ^{n} - 1,\,0} \right) } \end{aligned}$$

where $\prod$ denotes matrix convolution, ${\mathbb {I}}_{k}$ denotes the identity matrix of dimension $k \times k$, and ${\text {interspersecols}}$ is a function that takes as input an arbitrary matrix $\mathbf A$, a spacing *k* and an element *x*, and returns as output a new matrix, where the columns of $\mathbf A$ are interspersed with *k* columns of the specified element *x*.

Exemplar matrices for small values of *n* and *m* are given in Table 2.

**Table 2:** Matrices for conversion of isotopomer and mass fraction vectors

$\overline{\mathbf{IM }}_{n,m}$	$n=0$	$n=1$	$n=2$
$m=0$	$$\begin{bmatrix} 1 \end{bmatrix}$$	$$\begin{bmatrix} 1 \end{bmatrix}$$	$$\begin{bmatrix} 1 \end{bmatrix}$$
$m=1$	$$\begin{bmatrix} 1 \end{bmatrix}$$	$$\begin{bmatrix} 1&0\\ 0&1 \end{bmatrix}$$	$$\begin{bmatrix} 1&0&0&0\\ 0&1&1&0\\ 0&0&0&1 \end{bmatrix}$$
$m=2$	$$\begin{bmatrix} 1 \end{bmatrix}$$	$$\begin{bmatrix} 1&0&0\\ 0&1&0\\ 0&0&1 \end{bmatrix}$$	$$\begin{bmatrix} 1&0&0&0&0&0&0&0&0\\ 0&1&0&1&0&0&0&0&0\\ 0&0&1&0&1&0&1&0&0\\ 0&0&0&0&0&1&0&1&0\\ 0&0&0&0&0&0&0&0&1 \end{bmatrix}$$

Non-square, rectangular matrices are non-invertible. While every rectangular matrix has a Moore–Penrose pseudoinverse, their use, in this context, is incorrect, as we will now show with a pathological example.

Let A be a homonuclear moiety of two isotopic atoms, considering one isotopic tracer element, with two isotopes, distinguished by one mass shift, then conversion of isotopomer and mass fraction vectors is given by the linear equation

8a $$\begin{aligned} \begin{bmatrix} \begin{array}{c} {\text {A}}_{\left\{ {1,\,2} \right\} ,\,{\text {M}} + 0}\\ {\text {A}}_{\left\{ {1,\,2} \right\} ,\,{\text {M}} + 1}\\ {\text {A}}_{\left\{ {1,\,2} \right\} ,\,{\text {M}} + 2}\\ \end{array} \end{bmatrix} = \overline{\mathbf{IM }}_{2,\,1} \cdot \begin{bmatrix} \begin{array}{c} {\text {A}}_{\left( {0,\,0} \right) }\\ {\text {A}}_{\left( {0,\,1} \right) }\\ {\text {A}}_{\left( {1,\,0} \right) }\\ {\text {A}}_{\left( {1,\,1} \right) }\\ \end{array} \end{bmatrix}; \end{aligned}$$

and, to aid the example, assuming different values for each of the four isotopomer fractions of A, then

8b $$\begin{aligned} \begin{bmatrix} \begin{array}{c} 0.4\\ 0.5\\ 0.1\\ \end{array} \end{bmatrix} = \begin{bmatrix} \begin{array}{cccc} 1 & 0 & 0 & 0\\ 0 & 1 & 1 & 0\\ 0 & 0 & 0 & 1\\ \end{array} \end{bmatrix} \cdot \begin{bmatrix} \begin{array}{c} 0.4\\ 0.3\\ 0.2\\ 0.1\\ \end{array} \end{bmatrix}, \end{aligned}$$

which is correct, given the specified isotopomer fractions.

However, conversion between mass and isotopomer fraction vectors, using the Moore–Penrose pseudoinverse, is incorrect

8c $$\begin{aligned} \begin{bmatrix} \begin{array}{c} {\text {A}}_{\left( {0,\,0} \right) }\\ {\text {A}}_{\left( {0,\,1} \right) }\\ {\text {A}}_{\left( {1,\,0} \right) }\\ {\text {A}}_{\left( {1,\,1} \right) }\\ \end{array} \end{bmatrix} \ne \left( { \overline{\mathbf{IM }}_{2,\,1} } \right) ^{+} \cdot \begin{bmatrix} \begin{array}{c} {\text {A}}_{\left\{ {1,\,2} \right\} ,\,{\text {M}} + 0}\\ {\text {A}}_{\left\{ {1,\,2} \right\} ,\,{\text {M}} + 1}\\ {\text {A}}_{\left\{ {1,\,2} \right\} ,\,{\text {M}} + 2}\\ \end{array} \end{bmatrix}, \end{aligned}$$

since

8d $$\begin{aligned} \begin{bmatrix} \begin{array}{c} 0.4\\ 0.25\\ 0.25\\ 0.1\\ \end{array} \end{bmatrix} = \begin{bmatrix} \begin{array}{ccc} 1 & 0 & 0\\ 0 & 0.5 & 0\\ 0 & 0.5 & 0\\ 0 & 0 & 1\\ \end{array} \end{bmatrix} \cdot \begin{bmatrix} \begin{array}{c} 0.4\\ 0.5\\ 0.1\\ \end{array} \end{bmatrix}. \end{aligned}$$

### Logical conjunction and disjunction of isotopic labeling states

Let *m* be a non-negative integer that denotes the greatest neutron number, then logical conjunction is defined for pairs of members of the set ${\text {St}}_{m} \cup \left\{ \top \right\}$ that are either: equal, determinate and indeterminate, or indeterminate and determinate.

The Cayley table for the set ${{\text {St}}_{m} \cup {\left\{ {\bot , \top } \right\} }}$ under logical conjunction, with rows and columns permuted to facilitate this exposition, is9showing that the set ${\text {St}}_{m} \cup \left\{ {\bot , \top } \right\}$ is closed under logical conjunction, as are four of its cosets: $\left\{ {\bot } \right\} ,\left\{ {\top } \right\} ,\left\{ {\bot , \top } \right\}$, and ${\text {St}}_{m} \cup \left\{ {\bot } \right\}$; however, the set ${\text {St}}_{m}$, and by extension, the set ${\text {St}}_{m} \cup \left\{ \top \right\}$, is neither closed nor well-defined under logical conjunction; since, by definition, it does not contain $\bot$. The set ${{\text {St}}_{m} \cup {\left\{ {\bot , \top } \right\} }}$ is, therefore, a multi-valued logic, with *m* distinguished, mutually contradictory elements that are identified with neither logical true $\top$ nor logical false $\bot$.

Logical conjunction is, therefore, defined only for pairs of isotopomers that are pairwise equal; and is equivalent to multiplication of the corresponding isotopomer fractions; however, since logical conjunction is not defined for every pair of isotopomers, we cannot take the Cartesian product under logical conjunction of pairs of sets of isotopomers.

Logical conjunction is, therefore, defined only for pairs of cumomers that are pairwise: equal, determinate and indeterminate, or indeterminate and determinate; and it is equivalent to multiplication of the corresponding cumomer fractions. Since logical conjunction is defined for pairs of cumomers that are determinate for disjoint subsets of atoms, we can take the Cartesian product under logical conjunction of pairs of sets of cumomers that are determinate for disjoint subsets of atoms.

Logical disjunction is, of course, defined, for all isotopomers and cumomers, and it is equivalent to addition of the corresponding isotopomer and cumomer fractions.

### Decomposition of arbitrary biochemical reactions

A biochemical reaction is an identity of power-sets of isotopic atoms of virtual moieties, corresponding to the sets of all isotopic atoms on the left- and right-hand sides of the “arrow.” For example, the expression

10a $$\begin{aligned} {\text {A}}_{\left\{ {a, b} \right\} } + {\text {B}}_{\left\{ {c} \right\} } \overset{v}{\longrightarrow } {\text {C}}_{\left\{ {a} \right\} } + {\text {D}}_{\left\{ {b, c} \right\} } \end{aligned}$$

denotes a biochemical reaction with two reagents, A and B, two products, C and D, and a flux variable v. It is a representation of the identity

10b $$\begin{aligned} f\left( { \left( { {\mathbb {P}}\left( {\left\{ {a_{{\text {right}}}} \right\} } \right) \cup {\mathbb {P}}\left( {\left\{ {b_{{\text {right}}}, c_{{\text {right}}}} \right\} } \right) } \right) \setminus \emptyset } \right) = v \times f\left( { \left( { {\mathbb {P}}\left( {\left\{ {a_{{\text {left}}}, b_{{\text {left}}}} \right\} } \right) \cup {\mathbb {P}}\left( {\left\{ {c_{{\text {left}}}} \right\} } \right) } \right) \setminus \emptyset } \right) , \end{aligned}$$

where ${\mathbb {P}}$ denotes the power-set function, $\emptyset$ denotes the empty set, and the function *f* is a valid homomorphism, given the conditions that we have established.

Note the empty set is deliberately excluded from both power-sets. Doing otherwise would introduce a contradiction: $f\left( {\emptyset } \right) = v \times f\left( {\emptyset } \right)$.

Application of the power-set and set difference functions yields

10c $$\begin{aligned} f\left( { \left\{ {\left\{ {a_{{\text {right}}}} \right\} , \left\{ {b_{{\text {right}}}} \right\} , \left\{ {c_{{\text {right}}}}\right\} , \left\{ {b_{{\text {right}}}, c_{{\text {right}}}} \right\} } \right\} } \right) = v \times f\left( { \left\{ {\left\{ {a_{{\text {left}}}} \right\} , \left\{ {b_{{\text {left}}}} \right\} , \left\{ {a_{{\text {left}}}, b_{{\text {left}}}} \right\} , \left\{ {c_{{\text {left}}}} \right\} } \right\} } \right) ; \end{aligned}$$

and hence, the decomposition of the original identity is the system of identities

10d $$\begin{aligned} f\left( {\left\{ {a_{{\text {right}}}} \right\} } \right)&= v \times f\left( {\left\{ {a_{{\text {left}}}} \right\} } \right) \nonumber \\ f\left( {\left\{ {b_{{\text {right}}}} \right\} } \right)&= v \times f\left( {\left\{ {b_{{\text {left}}}} \right\} } \right) \nonumber \\ f\left( {\left\{ {c_{{\text {right}}}} \right\} } \right)&= v \times f\left( {\left\{ {c_{{\text {left}}}} \right\} } \right) \nonumber \\ f\left( {\left\{ {b_{{\text {right}}}, c_{{\text {right}}}} \right\} } \right)&= v \times \left( {f\left( {\left\{ {b_{{\text {left}}}} \right\} } \right) \star f\left( {\left\{ {c_{{\text {left}}}}\right\} } \right) } \right) \end{aligned}$$

where $\star$ denotes the monoidal combinator (for the codomain of the function *f*).

Replacement of each moiety by its isotopic labeling state, i.e., application of the function *f*, we obtain the system of identities

10e $$\begin{aligned} {\text {C}}_{\left\{ {1} \right\} }&= v \times {\text {A}}_{\left\{ {1} \right\} }\nonumber \\ {\text {D}}_{\left\{ {1} \right\} }&= v \times {\text {A}}_{\left\{ {2} \right\} }\nonumber \\ {\text {D}}_{\left\{ {2} \right\} }&= v \times {\text {B}}_{\left\{ {1} \right\} }\nonumber \\ {\text {D}}_{\left\{ {1,2} \right\} }&= v \times \left( {{\text {A}}_{\left\{ {2} \right\} } \star {\text {B}}_{\left\{ {1}\right\} }} \right) \end{aligned}$$

Thus, the problem of decomposition of representations of “biochemical reactions” is reduced to the decomposition of identities of power-sets of “isotopic atoms,” making no reference to the underlying chemistry, where “isotopic atoms” are, in practice, members of any countably infinite set for which equality can be established, e.g., the set of natural numbers.

Note that, unlike the “EMU decomposition” algorithm of Antoniewicz et al., our decomposition algorithm is exhaustive, runs in constant time and memory, has a worst-case computational complexity of ${\mathcal {O}}\left( {n^{2}} \right)$ with respect to the number of “isotopic atoms” *n*, and does not require backtracking.

### Formulation of flux balance equations

A biochemical reaction network is a set of biochemical reactions; and therefore, the decomposition of a biochemical reaction network is a set of systems of identities of isotopic labeling states.

From this point, formulation of the flux balance equations is purely mechanical:

1. Take the union of the set of systems of identities of isotopic labeling states;
2. Partition the resulting set by the number of isotopic atoms per identity;
3. Represent each partition as an adjacency matrix;
4. Optimize the adjacency matrices using any valid graph-theoretic method (optional, but highly recommended);
5. Formulate the flux balance equation for each adjacency matrix.

Adjacency matrices are representations of labeled, directed graphs. After permutation of the rows and columns, vectors of vertices (of the directed graphs) are of the form

11 $$\begin{aligned} a = \begin{bmatrix} \begin{array}{c} a_{1}\\ a_{2}\\ a_{3}\\ \end{array} \end{bmatrix}, \end{aligned}$$

where $a_{1}, a_{2}$ and $a_{3}$ denote, respectively, sub-vectors of source, intermediate and sink vertices, corresponding to substrates, intermediates and products; and, adjacency matrices are of the form

12 $$\begin{aligned} A = \begin{bmatrix} \begin{array}{ccc} 0 & 0 & 0\\ A_{2,1} & A_{2,2} & 0\\ A_{3,1} & A_{3,2} & 0\\ \end{array} \end{bmatrix}, \end{aligned}$$

where the element $A_{i,j} = \lambda$ denotes the identity $a_{j} = \lambda \times a_{i}$ for the coefficient $\lambda$.

Assuming a variational formulation of fluid dynamics for a bounded domain with a piecewise-smooth boundary and a general “mass” conservation law [15], flux balance equations are of the form

13 $$\begin{aligned} f\left( {\ldots } \right) = {\text {diag}}\left( {{\text {rowsum}}\left( { \begin{bmatrix} \begin{array}{ll} A_{2,1} &\quad A_{2,2}\\ A_{3,1} &\quad A_{3,2}\\ \end{array} \end{bmatrix} } \right) } \right) \cdot \begin{bmatrix} \begin{array}{l} a_{2}\\ a_{3}\\ \end{array} \end{bmatrix} - \begin{bmatrix} \begin{array}{cc} A_{2,1} &\quad A_{2,2}\\ A_{3,1} &\quad A_{3,2}\\ \end{array} \end{bmatrix} \cdot \begin{bmatrix} \begin{array}{l} a_{1}\\ a_{2}\\ \end{array} \end{bmatrix} \end{aligned}$$

where *f* is an arbitrary function, diag denotes a function that constructs a square matrix with the specified leading diagonal, and rowsum denotes a function that yields a column vector of the sums of the elements of each row of the specified matrix. (The steady-state hypothesis being $f\left( {\ldots } \right) \approx 0$).

On the right-hand side of (13), the first matrix is the mass lumping matrix for intermediate and sink vertices using the row sum technique; and, the second matrix is the discrete transport operator for source and intermediate vertices.

However, (13) is nonlinear, referring to the set of intermediates in the expressions of both influx and efflux; and therefore, is not in the required form. Rearranging the right-hand side of (13) using elementary algebra, we obtain

14 $$\begin{aligned} f\left( {\ldots } \right) = \left( { {\text {diag}}\left( {{\text {rowsum}}\left( { \begin{bmatrix} \begin{array}{cc} A_{2,1} & \quad A_{2,2}\\ A_{3,1} &\quad A_{3,2}\\ \end{array} \end{bmatrix} } \right) } \right) - \begin{bmatrix} \begin{array}{cc} A_{2,2} &\quad 0\\ A_{3,2} &\quad 0\\ \end{array} \end{bmatrix} } \right) \cdot \begin{bmatrix} \begin{array}{c} a_{2}\\ a_{3}\\ \end{array} \end{bmatrix} - \begin{bmatrix} \begin{array}{cc} A_{2,1}\\ A_{3,1}\\ \end{array} \end{bmatrix} \cdot a_{1} \end{aligned}$$

which is of the required form.

On the right-hand side of (14), the first matrix is the consistent mass matrix for intermediate and sink vertices; and, the second matrix is the discrete transport operator for source vertices only, i.e., the required form. Notice that the elements of the leading diagonal of the consistent mass matrix are positive. This is to ensure that the eigenvalues of the consistent mass matrix are non-negative [16].

Assuming the steady-state hypothesis, for example, we obtain

15 $$\begin{aligned} \left( { {\text {diag}}\left( {{\text {rowsum}}\left( { \begin{bmatrix} \begin{array}{cc} A_{2,1} &\quad A_{2,2}\\ A_{3,1} &\quad A_{3,2}\\ \end{array} \end{bmatrix} } \right) } \right) - \begin{bmatrix} \begin{array}{cc} A_{2,2} &\quad 0\\ A_{3,2} &\quad 0\\ \end{array} \end{bmatrix} } \right) \cdot \begin{bmatrix} \begin{array}{c} a_{2}\\ a_{3}\\ \end{array} \end{bmatrix} \approx \begin{bmatrix} \begin{array}{cc} A_{2,1}\\ A_{3,1}\\ \end{array} \end{bmatrix} \cdot a_{1} \end{aligned}$$

which is readily solved by inversion of the consistent mass matrix.

Thus, the problem of formulating cascade systems of flux balance equations for “biochemical reaction networks” is reduced to the construction and optimization of dependency networks of adjacency matrices, followed by the extraction of block matrices of predetermined dimensions, making no reference to the underlying chemistry.

### Practical usage of representations of detectable isotopic labeling states

In this manuscript, we have given constructive definitions of representations of isotopic labeling states of moieties. Our mathematical framework, independent of Chemistry, is based on the premise that every conceivable isotopic labeling state is (mathematically) representable. As such, we make no attempt to distinguish between (mathematically) conceivable and (experimentally) detectable isotopic labeling states. Instead, we assert that representations of undetectable isotopic labeling states be identified by logical false, i.e., that the probability of detection is =0%.

It remains to give examples of the practical usage of representations of detectable isotopic labeling states in the context of different experimentations: mass spectrometry (MS) and nuclear magnetic resonance (NMR) spectroscopy.

#### Mass spectrometry

Following an MS experiment, a post-processing function is applied to the experimentally obtained data in order to yield a representation of the isotopic labeling state of the detected fragments; typically, a mass distribution.

For example, in the case of electrospray mass ionization (ESI) and high-resolution MS experiments, where fragmentation is optional, the post-processing function may be the identity function. By contrast, in the case of gas chromatography mass spectrometry (GC-MS), experimentally obtained data is corrected with respect to the representation of the isotopic labeling state of the chemical derivatization agent, yielding an “underivatized” representation of the isotopic labeling state for each detected fragment.

Irrespective of the underlying MS experiment, each fragment is identified with an EMU.

For example, consider the cyano radical; molecular formula: ${\cdot}CN$. In a heteronuclear model, where the two isotopic atoms are, respectively, carbon and nitrogen, if an isotopically labeled cyano radical is detected using high-resolution MS, then the result is, by definition, a matrix of the probability of occurrence of each of the four possible combinations of detectable isotopes (viz., a mass fraction matrix).

**Table:** 

	$^{{\textsf {14}}}{\textsf {N}}$	$^{{\textsf {15}}}{\textsf {N}}$
^12^ ${\textsf{C}}$	$\mathrm {P}\left( {\left\{ {{\mathrm {C}} = 0} \right\} \cap \left\{ {\mathrm {N} = 0} \right\} } \right)$	$\mathrm {P}\left( {\left\{ {{\mathrm {C}} = 0} \right\} \cap \left\{ {\mathrm {N} = 1} \right\} } \right)$
^ 13^ ${\textsf{C}}$	$\mathrm {P}\left( {\left\{ {{\mathrm {C}} = 1} \right\} \cap \left\{ {\mathrm {N} = 0} \right\} } \right)$	$\mathrm {P}\left( {\left\{ {{\mathrm {C}} = 1} \right\} \cap \left\{ {\mathrm {N} = 1} \right\} } \right)$

Accordingly, the representation of each event corresponds to a specific isotopomer of the cyano radical;

**Table:** 

	$^{{\textsf {14}}}{\textsf {N}}$	$^{{\textsf {15}}}{\textsf {N}}$
^12^ ${\textsf{C}}$	${\cdot}CN_{\left( {0,\,0} \right) }$	${\cdot}CN_{\left( {0,\,1} \right) }$
^ 13^ ${\textsf{C}}$	${\cdot}CN_{\left( {1,\,0} \right) }$	${\cdot}CN_{\left( {1,\,1} \right) }$

which, in turn, are equivalent to isotopomers of EMUs of the cyano radical (precisely, the EMU that corresponds to the moiety of all atoms of the cyano radical).

**Table:** 

	$^{{\textsf {14}}}{\textsf {N}}$	$^{{\textsf {15}}}{\textsf {N}}$
^12^ ${\textsf{C}}$	${\cdot}CN_{\left\{ {1,\,2} \right\} ,\,\left( {0,\,0} \right) }$	${\cdot}CN_{\left\{ {1,\,2} \right\} ,\,\left( {0,\,1} \right) }$
^ 13^ ${\textsf{C}}$	${\cdot}CN_{\left\{ {1,\,2} \right\} ,\,\left( {1,\,0} \right) }$	${\cdot}CN_{\left\{ {1,\,2} \right\} ,\,\left( {1,\,1} \right) }$

Taking the trace of the anti-diagonals of the matrix, which corresponds to taking the logical disjunction of the subset of (heteronuclear) mass fractions that correspond to isotopically labeled moieties with the same number of additional neutrons, yields a vector; specifically, the mass fraction vector that is obtained by low-resolution MS of the same sample.

16 $$\begin{aligned} \begin{bmatrix} \begin{array}{c} {\cdot}{ CN}_{\left\{ {1,\,2} \right\} ,\,{\text {M}} + 0}\\ {\cdot}{ CN}_{\left\{ {1,\,2} \right\} ,\,{\text {M}} + 1}\\ {\cdot}{ CN}_{\left\{ {1,\,2} \right\} ,\,{\text {M}} + 2}\\ \end{array} \end{bmatrix} = \begin{bmatrix} \begin{array}{c} \mathrm {P}\left( {\left\{ {{\mathrm {C}} = 0} \right\} \cap \left\{ {\mathrm {N} = 0} \right\} } \right) \\ \mathrm {P}\left( { \left( { \left\{ {{\mathrm {C}} = 0} \right\} \cap \left\{ {\mathrm {N} = 1} \right\} } \right) \cup \left( { \left\{ {{\mathrm {C}} = 1} \right\} \cap \left\{ {\mathrm {N} = 0} \right\} }\right) } \right) \\ \mathrm {P}\left( {\left\{ {{\mathrm {C}} = 1} \right\} \cap \left\{ {\mathrm {N} = 1} \right\} } \right) \\ \end{array} \end{bmatrix} \end{aligned}$$

In this way, both low- and high-resolution MS data can be leveraged within the same mathematical framework.

#### Nuclear magnetic resonance

After Fourier transformation, the result of a NMR experiment (of arbitrary dimensionality) is a frequency-domain spectrum. If we assume, without loss of generality, that the integral for the resonance of a given “observed” nucleus of a given metabolite is equal to unity, then the proportional integral of a given NMR splitting pattern of a moiety of said metabolite is equal to the conditional probability of measuring a given isotopic labeling state of the “detected” nucleus whilst fixing the isotopic labeling state of the “observed” nucleus. Note that, in the context of a given moiety, the “detected” and “observed” nuclei need not be directly connected via a single chemical bond.

Consider Leucine (abbreviated: “Leu”), an amino acid of 6 Carbon atoms. The second Carbon atom is denoted ${\mathrm {C}}_{\alpha }$; whereas, the fifth and sixth Carbon atoms, indistinguishable by NMR, are both denoted ${\mathrm {C}}_{\delta }$.

The integral of the NMR splitting pattern

17a $$\begin{aligned} {\mathrm {C}}_{\alpha } - {\mathrm {C}}_{\delta } \end{aligned}$$

is equal to the conditional probability of the detected atom ${\mathrm {C}}_{\delta }$ being ^ 13^ ${\textsf{C}}$-labeled when the observed atom ${\mathrm {C}}_{\alpha }$ is also ^ 13^ ${\textsf{C}}$-labeled

17b $$\begin{aligned} \mathrm {P}\left( { \left\{ { {\mathrm {C}}_{\delta } = 1 } \right\} \vert \left\{ { {\mathrm {C}}_{\alpha } = 1 } \right\} } \right) , \end{aligned}$$

which, by definition, is equal to the quotient

17c $$\begin{aligned} \frac{ \mathrm {P}\left( { \left\{ { {\mathrm {C}}_{\delta } = 1 } \right\} \cap \left\{ { {\mathrm {C}}_{\alpha } = 1 } \right\} } \right) }{ \mathrm {P}\left( { \left\{ { {\mathrm {C}}_{\alpha } = 1 } \right\} } \right) }. \end{aligned}$$

Since there are two indistinguishable Carbon atoms, the probability of either or both of ${\mathrm {C}}_{\delta }$ being ^ 13^ ${\textsf{C}}$-labeled is equal to

17d $$\begin{aligned} \mathrm {P}\left( { \left( { \left( { \left\{ {{\mathrm {C}}_{\delta _{1}} = 0 } \right\} \cap \left\{ { {\mathrm {C}}_{\delta _{2}} = 1 } \right\} } \right) \cup \left( {\left\{ { {\mathrm {C}}_{\delta _{1}} = 1 } \right\} \cap \left\{ {{\mathrm {C}}_{\delta _{2}} = 0 } \right\} } \right) \cup \left( {\left\{ { {\mathrm {C}}_{\delta _{1}} = 1 } \right\} \cap \left\{ {{\mathrm {C}}_{\delta _{2}} = 1 } \right\} } \right) } \right) }\right) . \end{aligned}$$

Therefore, the result is equal to

17e $$\begin{aligned} \frac{ \left( { \mathrm {Leu}_{\left( {\top ,\,\top ,\,\top ,\,\top ,\,0,\,1} \right) } + \mathrm {Leu}_{\left( {\top ,\,\top ,\,\top ,\,\top ,\,1,\,0} \right) } + \mathrm {Leu}_{\left( {\top ,\,\top ,\,\top ,\,\top ,\,1,\,1} \right) } } \right) \times \mathrm {Leu}_{\left( {\top ,\,1,\,\top ,\,\top ,\,\top ,\,\top } \right) } }{ \mathrm {Leu}_{\left( {\top ,\,1,\,\top ,\,\top ,\,\top ,\,\top }\right) } }, \end{aligned}$$

which, given logical conjunction, is equal to

17f $$\begin{aligned} \frac{ \mathrm {Leu}_{\left( {\top ,\,1,\,\top ,\,\top ,\,0,\,1}\right) } + \mathrm {Leu}_{\left( {\top ,\,1,\,\top ,\,\top ,\,1,\,0}\right) } + \mathrm {Leu}_{\left( {\top ,\,1,\,\top ,\,\top ,\,1,\,1}\right) } }{ \mathrm {Leu}_{\left( {\top ,\,1,\,\top ,\,\top ,\,\top ,\,\top } \right) } }. \end{aligned}$$

Since cumomers of a metabolite are equivalent to isotopomers of EMUs of said metabolite, the final result is equal to

17g $$\begin{aligned} \frac{ \mathrm {Leu}_{\left\{ {2,\,5,\,6} \right\} ,\,\left( {1,\,0,\,1} \right) } + \mathrm {Leu}_{\left\{ {2,\,5,\,6}\right\} ,\,\left( {1,\,1,\,0} \right) } + \mathrm {Leu}_{\left\{ {2,\,5,\,6} \right\} ,\,\left( {1,\,1,\,1} \right) } }{\mathrm {Leu}_{\left\{ {2} \right\} ,\,\left( {1} \right) } }. \end{aligned}$$

## Discussion

Beginning with a single assumption (the quantization of nucleons in an atomic nucleus), we have formulated a mathematical framework for isotopic labeling that is independent of Chemistry.

Reclothing the theory of isotopic labeling with familiar concepts from Chemistry only serves to obscure its generality and rigor. Furthermore, imposing artificial constraints on the theory of isotopic labeling, motivated by Chemistry, rather than by Mathematics, is, obviously, incorrect. (As a corollary, imposing artificial constraints on the practice of isotopic labeling, such as limiting oneself to chemically and financially feasible experiments, is, obviously, correct).

Identifying the *true name* of the mathematical objects of the problem has allowed us to leverage the comparatively vast corpora of a diverse range of disciplines, including: Computer Science, Mathematics, Information Theory and Coding Theory. For example, we have shown that the set of punctured cumomers of a given moiety is the Reed–Muller spectral domain of the set of isotopomers of said moiety; and hence, that we can use the Reed–Muller transform matrices to convert isotopomer and punctured cumomer fraction vectors.

The isotopomer method, the most naïve representation of isotopic labeling state, is, clearly, flawed. Malloy et al. assume, incorrectly, that the “labeled” and “unlabeled” variants of isotopic tracer elements can be identified with the Boolean truth values. Instead, as we have shown, determinate isotopic labeling states are distinguished elements of a multi-valued logic, whose undistinguished elements are identified with contradiction $\bot$ and the indeterminate isotopic labeling state $\top$.

The cumomer method fairs slightly better. Weichert et al. do not recognize the complete set of cumomers of a given moiety. Instead, what they refer to as “cumomers” is the set of punctured cumomers with respect to $s = 0$. In contrast to the advice of the Mathematics community, they assume that the indeterminate isotopic labeling state is less than every determinate isotopic labeling state; and hence, the elements of Weichert et al.’s cumomer fraction vectors are out of order, making conversion between isotopomer and cumomer fraction vectors cumbersome in the general case. Furthermore, contrary to the advice of the Fluid Dynamics community, Weichert et al. assume that, in the flux balance equation, influx is positive and efflux is negative. Hence, in cumomer-based flux balance equations, the consistent mass matrix has non-positive eigenvalues.

We have shown that the EMU method can be “done” because the function that interprets a set of isotopic atoms (with determinate isotopic labeling states) as a mass distribution is a homomorphism, and because the set of mass distributions is a commutative monoid (when sets of isotopic atoms are pairwise disjoint).

From this new perspective, the cumomer method can be “done” for two reasons: because cumomer fraction vectors are a valid codomain of the homomorphism and EMU-based flux balance equations can be transformed into cumomer-based flux balance equations by elementary algebraic manipulations: replacement of flux variable coefficients by coefficient matrices; transposition of row vectors and subsequent construction of a block column vector; and, permutation of rows and columns.

Cumomer and mass fraction vectors are not the only “valid” interpretations. Zero dimensional, i.e., scalar, interpretations are possible, the simplest being the number of isotopic atoms per moiety. One dimensional, i.e., vector, interpretations include: cumomer fraction vectors, punctured cumomer fraction vectors and mass distributions. Two dimensional, i.e., matrix, interpretations, considering both the number of protons and neutrons of each moiety are also admissible. (In the flux balance equation, coefficient matrices are replaced by tensors).

We have shown that the “flux balance equation” is the “mass balance equation” of Fluid Dynamics, under the following assumptions:

- A bounded domain with a piecewise-smooth boundary;
- A unit volume;
- An incompressible fluid;
- Mass lumping approximated by the row-sum technique; and,
- A distance metric that is constant for all pairs, i.e., that all “stuff” in a compartment is equally, infinitely close.

This suggests that the flux balance equation could be derived from Fluid Dynamics under much more general assumptions. In the forwards direction, this technology transfer could enable the translation of Systems Biology models into Fluid Dynamics models, and subsequently, the simulation of Systems Biology models using Fluid Dynamics tools. In the backwards direction, establishing a dictionary could enable Fluid Dynamics concepts, theorems and results to be utilized by Systems Biology researchers. For example, what are the “translations” of laminar flow and friction? Moreover, can a biological reaction network be embedded in an arbitrary manifold, thereby allowing the model to account for the spatial characteristics of the compartment?

## Conclusions

The contributions of this article are as follows:

- We have rigorously defined the concept of an isotopic labeling state of an arbitrary moiety, consisting of an arbitrary number of isotopic atoms, with an arbitrary number of isotopes per isotopic tracer element, distinguished by an arbitrary number of mass shifts.
- We have reformulated the isotopomer, cumomer and EMU methods in terms of our new formulation of isotopic labeling states; demonstrating both the decomposition of arbitrary biochemical reaction networks and the construction of the corresponding flux balance equations.
- We have given matrices for the conversion of arbitrary isotopomer, cumomer and mass fraction vectors; recognizing that the set of punctured cumomers of a given moiety (with respect to any determinate isotopic labeling state) is the Reed–Muller spectral domain of the corresponding set of isotopomers.

With this work, it is now possible to simulate the isotopic labeling states of metabolites in completely arbitrary biochemical reaction networks.

An immediate application of this work is the development of software systems that are informed of all mathematical relationships between the mathematical objects.

We expect that this work will be incorporated into future implementations of Systems Biology software systems.

### Future work

 The following research questions are unanswered by this study and are left for future work:

- How should the new capability, determination of isotopic labeling states of heteronuclear moieties, be leveraged? For example, is it more computationally efficient and/or numerically precise to fit metabolic flux variables to experimentally obtained measurements of hetero- rather than homonuclear moieties?
- This study assumes that isotopic labeling states of atoms in a given moiety are mutually independent. Is it possible to provide a formulation where isotopic labeling states are conditionally dependent?
- In the context of Radiochemistry, can the new formulation be used to determine isotopic labeling states of moieties in the presence of radioactive decay and/or neutron sources?
